# Systematic review of pharmacological treatments in fragile X syndrome

**DOI:** 10.1186/1471-2377-9-53

**Published:** 2009-10-13

**Authors:** Jose-Ramon Rueda, Javier Ballesteros, Maria-Isabel Tejada

**Affiliations:** 1 Department of Preventive Medicine and Public Health, University of the Basque Country, Barrio Sarriena S/N, Leioa 48940, Spain; 2 Department of Neurosciences, University of the Basque Country, Barrio Sarriena S/N, Leioa 48940 and CIBER in Mental Health (CIBERSAM), Spain; 3 Molecular Genetics laboratory, Department of Biochemistry, GIRMOGEN, Cruces Hospital, Plaza de Cruces s/n, Barakaldo 48903, Spain

## Abstract

**Background:**

Fragile X syndrome (FXS) is considered the most common cause of inherited mental retardation. Affected people have mental impairment that can include Attention Deficit and/or Hyperactivity Disorder (ADHD), autism disorder, and speech and behavioural disorders. Several pharmacological interventions have been proposed to treat those impairments.

**Methods:**

Systematic review of the literature and summary of the evidence from clinical controlled trials that compared at least one pharmacological treatment with placebo or other treatment in individuals with diagnosis of FXS syndrome and assessed the efficacy and/or safety of the treatments. Studies were identified by a search of PubMed, EMBASE and the Cochrane Databases using the terms fragile X and treatment. Risk of bias of the studies was assessed by using the Cochrane Collaboration criteria.

**Results:**

The search identified 276 potential articles and 14 studies satisfied inclusion criteria. Of these, 10 studies on folic acid (9 with crossover design, only 1 of them with good methodological quality and low risk of bias) did not find in general significant improvements. A small sample size trial assessed dextroamphetamine and methylphenidate in patients with an additional diagnosis of ADHD and found some improvements in those taking methylphenidate, but the length of follow-up was too short. Two studies on L-acetylcarnitine, showed positive effects and no side effects in patients with an additional diagnosis of ADHD. Finally, one study on patients with an additional diagnosis of autism assessed ampakine compound CX516 and found no significant differences between treatment and placebo. Regarding safety, none of the studies that assessed that area found relevant side effects, but the number of patients included was too small to detect side effects with low incidence.

**Conclusion:**

Currently there is no robust evidence to support recommendations on pharmacological treatments in patients with FXS in general or in those with an additional diagnosis of ADHD or autism.

## Background

Fragile X syndrome, caused by a large expansion of a CGG trinucleotide repeat within the FMR1 gene located on chromosome X (q27.3) [1], is considered the most common cause of inherited mental retardation. It has been estimated that FXS syndrome affects approximately 1 in 4,000 males and 1 in 8,000 females [2]. However those estimates are based on population projections from cohorts of children with special education needs and thus they could underestimate the extent of clinical involvement as some individuals affected by the behavioural, emotional and/or learning disabilities of FXS could have IQs in the normal or borderline range [3,4].

People with FXS have mental dysfunction that normally includes some degree of mental retardation, ranging from severe to mild. The dysfunction can also include Attention Deficit and/or Hyperactivity Disorder (ADHD) [5,6], autism disorder or the autism spectrum disorder [7,8] and speech and behavioural disorders, such as anxiety-related symptoms including Obsessive Compulsive Disorder-like and perseverative behaviours, emotional lability and aggressive or self-aggressive behaviours.

The rationale underlying pharmacological treatments in persons with FXS is diverse and it has been extensively covered elsewhere [9-11]. After the discovery of FXS it was observed that chromosomes from people with fragile X needed to be cultured in solutions deficient in folic acid in order to reveal the defect and it was thought that maybe individuals with fragile X syndrome had a lack of folic acid in their bodies, or were unable to make optimal use of the folic acid they had. Therefore, it was argued that supplementing their dietary intake might remediate adverse developmental and behavioural effects of the condition and the first therapeutic trials focussed primarily on folic acid. More recently, pharmacological treatment strategies have been focussed mainly as supportive strategies designed to maximize social functioning. As behaviour in FXS can significantly impact functionality, symptom-based treatment of the most problematic behaviours in the individual with FXS can be quite helpful [12]. As many people with FXS had additional diagnosis of ADHD, stimulants like methylphenidate and dextroamphetamine-already efficacious in non FXS patients-have been assessed in people affected with FXS. In order to avoid some of the side effects observed in treatment with stimulants other researchers looked for a non-stimulant pharmacological treatment for ADHD symptoms in people with FXS and tested L-acetylcarnitine, a product that had been shown to inhibit *in vitro *the cytogenetic expression of the FXS-associated fragile site FRAXA [13]. CX 156, an AMPA receptor-positive modulator has been evaluated to study its effects on cognitive disability in people with FXS, given that recent understanding of defects in synaptic plasticity in the *fmr1 *knock-out KO mouse had led to the proposal of several pharmacological targets in FXS to attempt to normalize synaptic connectivity, including AMPA receptor activation [14].

To our knowledge, while several recently published articles include narrative reviews of this topic [10-12,15,16], to date no comprehensive systematic review of the safety and efficacy of pharmacological interventions in people with FXS has been published.

## Methods

### Objective

To systematically review the evidence on the efficacy and safety of pharmacotherapy in the treatment of people with FXS.

#### Inclusion criteria

We included all Clinical Controlled Trials (CCTs), randomized or not, that fulfil all the following criteria: (i) compared at least one pharmacological treatment with placebo or other treatment, (ii) included people with a diagnosis of FXS, (iii) assessed efficacy and/or safety of the treatments, and (iv) included as a main outcome results on psychological and social performance measured by standardized or validated scales.

Studies that assessed the impact of different medications in people with fragile X pre-mutation related syndromes, such as Fragile X-associated tremor/ataxia syndrome (FXTAS) or premature ovarian failure, were not included here.

### Search strategies

Electronic searches were performed in PubMed using "fragile X AND treatment" text words and the following limits: "Humans, Clinical Trial, Meta-Analysis, Practice Guideline, Randomized Controlled Trial, Review", without language restriction. EMBASE was searched via Ovid SP using "fragile X AND treatment" text words and limited to studies in humans. CENTRAL and the other Cochrane Library databases were searched using "fragile X" text words. The electronic searches were closed in March 2009 and complemented with reference lists from the trials and reviews retrieved. Two reviewers screened each abstract and decided which studies fulfilled the inclusion criteria.

### Outcomes selected

For the purpose of this review only outcomes measured by standardized instruments, regarding safety for the patients and clinical efficacy were taken into account. For efficacy, outcome domains included are those related with intelligence and behavioural, emotional and/or learning capabilities.

Certain measures like percentages of fragile X cells or folate levels in the blood, were assessed in some CCTs, but will not be presented here due to their unclear clinical implications.

### Data extraction and evaluation of methodological quality of the studies

Data on the main characteristics of the clinical trials included in this review were extracted independently by 2 reviewers, disagreements being solved by consensus. Methodological quality of the studies was assessed using the Cochrane Collaboration's criteria [17], including the following domains to assess the risk of bias: allocation sequence generation; allocation concealment; blinding of participants, personnel and assessors; incomplete outcome data; selective outcome reporting.

### Statistical methods

Even though the initial intention was to perform a statistical integration of the results when there was more than one study on any specific drug and outcome, this was precluded by clinical heterogeneity of the populations, scarcity of numerical data and the huge variability of outcome measures reported among studies.

## Results

### Literature search

**Figure 1:**
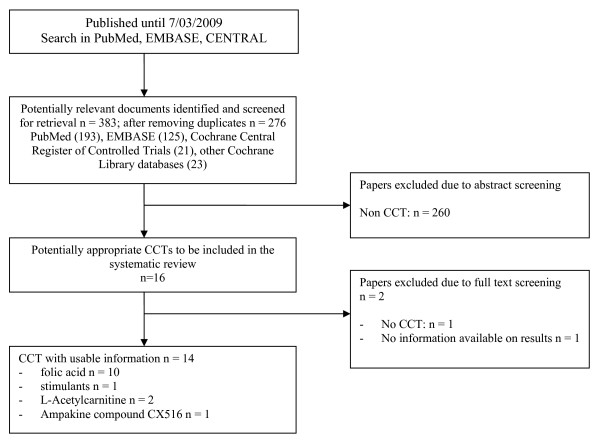
**Bibliographic review**. Flow diagram.

The literature searches identified a total of 383 documents which was reduced to 276 after removing duplicates. After screening the abstracts, 16 full text documents were retrieved and assessed. Two studies were obtained from the text and references of other studies. One CCT study on folic acid, published only as an abstract for a professional meeting, was not included in this review due to absence of data concerning results [18]. Another study that assessed the effects of propanolol on stereotyped behaviours in a single person was considered as a case study, not a CCT [19]. Finally, 14 studies fulfilled the criteria for inclusion. More detailed information is given in figure 1, where the QUORUM flow diagram [20] is presented.

### Efficacy and safety of pharmaceutical interventions

In the following sections, results and discussion will be presented separately for the different interventions. Tables 1, 2 and 3 present the information regarding the main characteristics of trials and main results.

**Table 1:** Characteristics of studies

	**Folic acid (FA)**		
***Reference***	***Participants and Follow-Up Period***	***Methods***	***Interventions***

Brown 1984 [21]	N = 2 brothers, 10.5 and 18.8 years. Setting: in-patients. *FUP*: 16 days	R, CO, DB. AASG: no; AAC: unclear; AB: unclear; IDAA: yes; FSR: yes.	FA 1,6 mg/kg intravenous *vs *Placebo

Brown 1986 [22]	N = 5 males, 8 to 26 years. 3 autism *FUP*: 9 months	R, CO, DB. AASG: unclear; AAC: unclear; AB: unclear; IDAA: yes; FSR: yes.	FA 250 mg/day *vs *Placebo

Carpenter 1983 [23]	N = 4 males, 2 to 10 years. IQs: 45 to 50. 2 autistic behaviour. *FUP: 6 *months	NR, CO, DB. AASG: no; AAC: unclear; AB: unclear; IDAA: unclear; FSR: unclear.	FA 10 mg/day *vs *Placebo

Fisch 1988 [24]	N = 6 males, 3.5 to 15 years. 3 autistic, 3 mental retardation only (borderline to severe). *FUP*: 12 months	R, CO, DB, SA. AASG: unclear; AAC: unclear; AB: unclear; IDAA: yes; FSR: yes.	FA 10 mg/day *vs *Placebo

Froster-Iskenius 1986 [29]	N = 10 males, 15 to 54 years. IQ: 21 to 60. *FUP*: 4 months	R, P, DB. AASG: unclear; AAC: unclear; AB: no; IDAA: yes; FSR: yes.	FA 10 mg/day *vs *0.0015 mg of folic acid/day, 2 months and 2 months with folic acid, 10 mg/day

Gillberg 1986 [30]	N = 4 males, 6 to 14 years. IQ: 39 to 64. All autism, 3 epilepsy. *FUP*: 9 months	NR, CO, DB, SA. AASG: no; AAC: unclear; AB: unclear; IDAA: yes; FSR: yes.	FA 5 mg/day *vs *Placebo

Hagerman 1986 [25]	N = 25 males, 1 to 31 years. IQ: 39 to 82. Many of the younger ones in special education program with language and occupational therapy. Many adults use phenothiazines. *FUP*: 12 months	R, CO, DB, SA. AASG: yes; AAC: unclear; AB: yes; IDAA: yes; FSR: yes.	FA 10 mg/day *vs *Placebo

Madison 1986 [26]	N = 3 males, 3, 8 and 12 years old. Moderate mental retardation. *FUP*: 11 weeks	NR, DB. AASG: no; AAC: unclear; AB: unclear; IDAA: yes; FSR: yes.	No drug-no placebo (20 days). Placebo (13-45 days). Folic acid 10 mg/day (10-43 days)

Rosenblatt 1985 [28]	N = 2 monozygous male twins, 14 years old, mentally retarded. *FU*: 12 months	NR, CO. AASG: no; AAC: unclear; AB: unclear; IDAA: yes; FSR: yes.	FA 5 mg/day *vs *Placebo

Strom 1992 [27]	N = 21 males, 2-22 years, mean 8.3 years. *FUP*: 24 weeks	R, CO, DB, SA. AASG: unclear; AAC: unclear; AB: unclear; IDAA: yes; FSR: yes.	FA 15 mg/day *vs *Placebo

	**Ampakine compound CX516**		

Berry-Kravis 2006 [14]	N = 49. 11 female, 18-49 years. IQ: 36 to75, mean 43; 12 ≥50. Autism 21, spectrum: 4. 27 on psychoactive medication. *FUP*: 4 weeks	Phase II, R, P, DB, SA. AASG: yes; AAC: yes; AB: yes; IDAA: yes; FSR: yes.	Ampakine compound CX516 600-900 mg/day *vs *Placebo

	**Dextroamphetamine Methylphenidate**		

Hagerman 1988 [5]	N = 15, 2 female. 3.8-11.8 years. IQ: 29 to 77 (3 IQ >70). All attentional problems. 40% on stimulants. *FUP*: 3 weeks	R, CO, DB, SA. AASG: unclear; AAC: unclear; AB: yes; IDAA: yes; FSR: yes.	Dextroamphetamine 2 mg/kg/day *vs *Methylphenidate 0.6 mg/kg/day *vs *Placebo

	**L-Acetylcarnitine**		

Torrioli 1999 [32]	N = 20 males, 6-13 years. IQ:30 to 69 Hyperactive behaviour. *FUP*: 12 months	R, P, DB, SA. AASG: unclear; AAC: unclear; AB: unclear; IDAA: unclear; FSR: yes.	L-Acetylcarnitine 100 mg/kg/day *vs *Placebo

Torrioli 2008 [13]	N = 63 males, 6-13 years, all with Attention Deficit Hyperactivity Disorder. All extra care and stimulation at school and home, and visits by neuropsychiatrists. *FUP*: 12 months	Phase II, R, P, DB, SA. AASG: unclear; AAC: unclear; AB: unclear; IDAA: yes; FSR: yes.	L-Acetylcarnitine 20-50 mg/kg/day *vs *Placebo

* AAC: adequate allocation concealment; AASG: adequate allocation sequence generation; AB: adequate blinding of participants, personnel and assessors; CO: crossover; DB: double blind; FSR: free of selective outcome reporting; FUP: follow up period; IDAA: incomplete data adequately addressed; N: number of patients; NR: non randomized; P: parallel; R: randomized; SA: statistical analysis presented.

**Table 2:** Outcome measures and main results of studies on folic acid

***Reference***	***Measures of interest: assessment method***	***Main Results***
Brown 1984 [21]	Psychological: Stanford-Binet, Peabody Picture Vocabulary Test Revised (PPVTR) Leither International Performance Scale of non-verbal performance. Scoring by researchers and parents for eye contact, appropriate social smiling, communicative speech and hyperventilating.	Stanford Binet IQ scores did not change significantly. PPVT-R scores appeared unrelated to folic acid treatment. Leither test of non-verbal performance showed improvement in one subject, from 3.0 to 5.6, but not in the other (no more details in article). Statistical testing not presented. Safety: no side effects.

Brown 1986 [22]	Leiter or Wechsler IQ tests. Autistic Descriptors Checklist (ADC): parental reporting rating checklist. Alpern-Ball test.	Changes of performance ratings not correlated with treatment. Pooled data not provided, only person by person. Statistical testing not presented. Safety: nothing reported.

Carpenter 1983 [23]	Psychological, speech and language, and psychiatric evaluations but not specified.	No measurable improvement in speech, language or intellectual abilities were found during therapy. Numeric data not provided. Statistical testing not presented. Safety: nothing reported. Note: Data from abstract.

Fisch 1988 [24]	Autistic Descriptors Checklist (ADC). Vineland Adaptative Behaviour Scale (VABS).	No statistically significant improvements in periods of folic acid treatment, neither in prepubescent nor in adolescent patients. Pooled data not provided, only person by person. Safety: nothing reported.

Froster-Iskenius 1986 [29]	Coloured Progressive Matrices test (CPM). Test for fine motor co-ordination, concentration and comprehension.	No improvement in concentration, fine motor co-ordination or comprehension in adults, but may have some effect in children. Pooled data not provided, only person by person. Statistical testing not presented. Safety: nothing reported.

Gillberg 1986 [30]	Autism Behaviour Checklist (ABC) and other checklist questionnaires pertaining to autism developed by authors. Parents "unstructured" diaries on child overall behaviour and language skills.	Mean ABC scores did not differ in a statistically significant way between placebo and folic acid periods. Numerical detailed information not provided. Safety: nothing reported.

Hagerman 1986 [25]	Speech and language testing: Peabody Picture Vocabulary Test (PPVT), Test of Language Development (TOLD) and an apraxia battery. Psychological testing: Yale Revised Developmental Schedules, Stanford-Binet, Leiter International Scale. Behavioural assessments: Autism Behavior Checklist (ABC) of the Autism Screening Instrument for Educational Planning (ASIEP) and the Childhood Autism Rating Scale (CARS).	No statistical differences (Wilcoson Rank Sum Test) between placebo and folic acid in: change in IQ, language (PPVT, TOLD, apraxia battery) behavioural areas, CARS, ABC. Psychological testing: IQ scores statistically significant improvement in prepuberal males while being treated with folic acid (one tailed p = 0.014; two tailed p = 0.028). Safety: nothing reported.

Madison 1986 [26]	Memory skills: Automated Device for the Assessment of Memory (ADAM); Verbal recall of objects and number series. Compliance and behavioural appropriateness.	No evident change in memory skills, compliance and behavioural appropriateness during treatment phase. Pooled data not provided, only person by person. Statistical testing not presented. Safety: nothing reported.

Rosenblatt 1985 [28]	Wechsler Intelligence Scale for Children-Revised (WISC-R) Peabody Picture Vocabulary Test Form M (PPVT), Goodenough-Harris Drawing Test (G-H), Rey Children's Word List, Token Test of Auditory Comprehension, Producing Names on Confrontation (test 8 of Clinical Evaluation of Language functioning (CELF), Boston Naming Test, Developmental Test of Visual-Motor integration (DTVMI), Wide Range Achievement Test (WRAT), reading Comprehension (Barnell Loft Multiple Skills Series Al) and Domino Pattern Counting Task. Parent's Questionnaire, Teacher Behavior Checklist and a Checklist of Problem Behaviors.	

Strom 1992 [27]	Cognitive function and behavioural level: Vineland Adaptive Behavioral Scales, Peabody Picture Vocabulary Test-Revised (PPVT-R), Conners' Parent and Teaching Rating Scales, the ADD-H: Comprehensive Teacher's Rating Scales (ACTeRS), and a questionnaire designed by the researchers	No statistically significant differences between the folic acid and placebo phases of the study in any outcome measurement instrument: Vineland (51.0 *vs *50.9); PPVT-R (55.4 vs 59.2), Conners' (15.5 *vs *13.4). Safety: minor side effects (transient problems with diarrhea, sleep delays, mood swings.

**Table 3:** Outcome measures and main results of studies not on folic acid

	**Ampakine compound CX516**	
***Reference***	***Measures of interest: assessment method***	***Main Results***

Berry-Kravis 2006 [14]	Primary outcome, memory domain: Visual Memory and Visual Sequential Memory Subtests of the Test of Visual--Perceptual Skills (TVPS), the Memory for Words Subtest of the Woodcock-Johnson Tests of Cognitive Ability--Revised and the Repeatable Battery for the Assessment of the Neuropsychological Status (RBANS). Secondary outcome measures. Attention/Executive Function Domain: SNAP IV. Language Domain: Peabody Picture Vocabulary Test-III, Forms A and B (PPVT-III) or the Clinical Evaluation of Language Fundamentals-3 (CELF-3). Behavioral Domain: Autism Diagnostic Observation Scale (ADOS), the Gilliam Autism Rating Scale (GARS), the Childhood Autism Rating Scale (CARS), a clinician-rated scale to evaluate modification of autistic features, the ABC-C, and the behaviour component of CGI - Improvement (CGI-I) and VAS. Clinical Cognitive Improvement Measures: VAS (caregiver rated) for cognition and the subject's chosen task (described above) and CGI-I (clinician rated) for cognition and the task. Adverse events: Aberrant Behavior Checklist - Community Edition (ABC-C).	Wilkoson rank-sum test performed. No significant improvement in memory, the primary outcome measure, or in secondary measures of language, attention/executive function, behaviour, and overall functioning in CX516-treated subjects compared to placebo. There were minimal side effects, no significant changes in safety parameters, and no serious adverse events. There was a 12.5% frequency of allergic rash in the CX516 group and 1 subject developed a substantial rash.

	**Dextroamphetamine Methylphenidate**	

***Reference***	***Measures of interest: assessment method***	***Main Results***

Hagerman 1988 [5]	Conners' Abbreviated Parent-Teacher Questionnaire. ADDH: Comprehensive Teacher Rating Scale (ACTeRS). Behavioural observation. Measure of movement: Large scale integrated sensor actometer (LSI). Delay task. Vigilance task.	Paired t tests performed. Compare to placebo clinical response in two thirds of patients, but no statistically significant difference between amphetamine and placebo for any of the ADHD measures, except for the improvement seen on the ACTeRS scale completed by the teacher. Social skills factor and improvements in attention significantly better with methylphenidate (mostly in mildly retarded persons) but not with amphetamine. Safety: significantly more side effects while taking amphetamine, mainly mood lability and irritability.

	**L-Acetylcarnitine**	

***Reference***	***Measures of interest: assessment method***	***Main Results***

Torrioli 1999 [32]	Wechsler Intelligence Scale for Children-Revised (WISC-R); the Bender Gestalt test; and the Conners' Abbreviated Parent-Teacher Questionnaire.	Non-parametric Wilcoson independent-sample test performed. No statistically significant difference between L-Acetylcarnitine and placebo in Wechsler Scale and Bender Gestalt tests and Conners' questionnaire completed by teachers. The Conners' Abbreviated Parent questionnaire showed a significant reduction (P = 0.0065) of hyperactive behaviour at one year in the LAC-treated subjects. Safety: no side effects noted in LAC group.

Torrioli 2008 [13]	Conners' Global Index-Parents (CGI-P) and Conners' Global Index-Teachers (CGI-T). Vineland Adaptive Behavior Scales-Survey Form (VABS) to evaluate adaptive behaviour, four domains: communication, daily living skills, socialization, and motor skills. Wechsler Intelligence Scale for Children-Revised (WISC-R). Side effects.	T tests and repeated measures multivariate analysis using the general linear model were performed. Statistically significant stronger reduction of hyperactivity and improvement of social behaviour in patients treated with LAC, compared with the placebo group, in CGI-P y VABS. Both groups improved their behaviour, showing that psychosocial intervention has a significant therapeutic effect. Safety: no side effects in LAC group.

### Folic acid

We found 10 published CCTs that assessed the efficacy and safety of folic acid treatment in people with FXS. The first studies were published in 1983 and the last one in 1992. Seven of the studies were performed in the USA [21-27], one in Canada [28], one in Germany [29] and one in Sweden [30].

The number of participants in the studies was in general very small, ranging from two to 25 individuals, including overall 82 people. All these participants in the studies were male and their ages ranged from 1.5 to 54 years. The degree of mental retardation varied from borderline to severe and four studies included patients with an additional diagnosis of autism or autistic behaviour [22-24,30,31]. Just one study was performed on an in-patient basis [21] and the duration of the follow-up in the studies ranged from sixteen days to twelve months.

Doses of folic acid varied, ranging from 5 mg/day to 250 mg/day, 10 mg/day being the most commonly used dosage, as presented in six of the studies. Nine of the studies used placebo as the control intervention and one study used a control preparation with a dose less than 0.0015 mg/day of folic acid [29].

None of the trials presented a parallel design. Nine of the ten studies were crossovers and in one study all patients took placebo first and then folic acid [26].

In five of the clinical trials the order of medications was randomised, but only one of them is known to have used an acceptable method of randomisation [25]. In fact, the method of randomisation was not stated in the other four reports [21,24,27,29]. Only one study could be classified as being of overall good methodological quality and low risk of bias [25].

Overall, the studies did not find significant improvement in outcomes assessed in periods taking folic acid compared to placebo. Only one study found a statistically significant improvement in an outcome variable in the period of folic acid treatment but this was found in a subgroup of eight patients (improvement of IQ in prepuberal children) [25].

The study in which all patients had an additional diagnosis of autism included only four patients and did not find significant differences between folic acid and placebo in the assessed outcomes; in particular, mean scores of the Autism Behaviour Checklist did not differ significantly between placebo and folic acid periods [30]. Two other studies that included some patients with an additional diagnosis of autism showed some improvements measured by the Autistic Descriptors Checklist, but they did not carry out statistical analysis [22,24]. Another study included two patients with an additional diagnosis of autistic behaviour, but did not provide separate data on them [23]. Overall the quality of reporting of quantitative data was poor and did not allow us to extract reliable effect sizes.

Regarding safety only two of the studies reported on side effects; one of them found no side effects [21] and the other found only minor transient side effects [27].

### Dextroamphetamine and methylphenidate

One crossover, randomised, double blind trial assessed the efficacy of dextroamphetamine and methylphenidate versus placebo in 15 children, two of them women, with FXS and an additional diagnosis of Attention Deficit Hyperactivity Disorder (ADHD) [5]. The length of the follow-up was three weeks, but each intervention lasted only one week. The authors reported that, compared to placebo, there was a clinical response in two thirds of patients, but no statistically significant difference between amphetamine and placebo for any of the ADHD measures, except for the improvement seen on the ACTeRS scale completed by the teacher. However, the article does not present enough data to calculate appropriate effect sizes. The social skills factor and improvements in attention were significantly better with methylphenidate (mostly in mildly retarded individuals) but not with amphetamine. Significantly more side effects appeared while taking amphetamine, mainly mood lability and irritability.

### L-Acetylcarnitine (LAC)

Two double-blind trials have assessed the safety and efficacy of LAC in boys with FXS and an additional diagnosis of ADHD [13,32]. Both of these were randomised placebo-controlled and used a parallel design.

The first study included 20 patients and compared LAC, in a dose of 100 mg/kg/day versus placebo, and found no significant difference between LAC and placebo on the Wechsler Scale or in the Bender Gestalt test and Conners' questionnaire completed by teachers. However, the Conners' Abbreviated Parent-Teacher questionnaire completed by parents showed a significant reduction (Hedges g effect size = -3.94; SE = 0.91) of hyperactive behaviour at the end of the study in the LAC-treated subjects [32].

The second study, classified by the authors as a Phase II study, involving eight centres in three European countries, compared LAC, at doses of 20-50 mg/kg/day versus placebo, in 63 patients [13]. All the patients received extra care and stimulation both at school and at home, and made regular visits to neuropsychiatrists who prompted a revision of the support activities that children received (speech therapy, physical therapy, occupational therapy, or other modality). The authors report that both groups improved their behaviour, showing that psychosocial intervention has a significant therapeutic effect. Statistically significant stronger reduction of hyperactivity and improvement of social behaviour was observed in patients treated with LAC, compared with the placebo group, on Conners' Global Index-Parents (CGI-P) (Hedges g effect size = -0.30; SE = 0.28) and Vineland Adaptive Behavior Scales - Survey Form (VABS) (Hedges g = 0.52 and 0.65; SE = 0.29 and 0.29). They also reported no significant side-effects in LAC group.

### Ampakine compound CX516

One phase II parallel, randomised, double-blind, placebo-controlled clinical trial of four weeks of duration, assessed efficacy and safety of ampakine compound CX516 versus placebo in 49 people with FXS, 27 of them taking concomitant psychoactive medication [14]. Twenty one patients had an additional diagnosis of autism and four of autism spectrum.

This study found no significant improvement in memory, the primary outcome measure, or in secondary measures of language, attention/executive function, behaviour, and overall functioning in CX516-treated subjects compared to placebo. There were minimal side effects, no significant changes in safety parameters, and no serious adverse events.

## Discussion

In this systematic review we have not found reliable and solid evidence to support the recommendation of any specific medication for people with FXS. There are different reasons to justify this conclusion. Some reasons apply to all the treatments studied and others apply only to a specific intervention.

It is general the problem related with the small sample sizes of the studies, which can result in problems in two areas: increased probability of type 2 errors (positive effects found in some studies could have been statistically significant if the samples had been larger) and inadequate size to detect side effects with low incidence. It is also striking the fact that very few women have been included in the studies, which probably can be explained by the previously extended belief that the disease was a problem only in males. In relation to the small numbers of patients, and also to the scarcity of studies, it must be taken into account that FXS is a relatively new disease, the specific genetic mutation was identified in 1991, and that its prevalence is low, being included into the category of the so-called "rare diseases". It is understandable that few research groups, and with limited resources, have been willing and able to carry out clinical trials on people with FXS. It also takes time until research from animal models is translated to assessment in human beings [33].

Regarding to the design of the studies and the assessment of their potential risk of bias, only a few of them could be classified as being of acceptable or good methodological quality, although it must be taken into account that only two studies were published after the CONSORT statement on standards for reporting trials [34]. Indeed, many of the publications provide insufficient information to allow critical assessment of the methodological quality of the trials and proper evaluation of the risk of bias. So it is difficult to know whether a particular study is methodologically flawed in the design or whether the reporting of the methods is incomplete.

Concerning specifically folic acid, it must be taken into account that apart from one study [26] the trials that assessed folic acid versus placebo were crossover studies and that the possibility of a 'carry-over' of treatment effect from one period to the next cannot be discarded. A carry-over effect means that the observed difference between the treatments depends upon the order in which they were received; hence the estimated overall treatment effect will be affected (usually underestimated, leading to a bias towards the null) [35]. One study showed positive statistically significant results on IQ measurements in a small group of 8 prepuberal children, but not in older patients [25]. In younger children it cannot be discarded that normal neuro-developmental changes could explain impressive improvements in some children in periods of time as short as six months; and in that study most of the children with the largest positive effects also had significant improvements in the placebo phase.

Regarding stimulants, the only RCT that assessed their effect in 15 children with FXS also diagnosed with ADHD [5] found some positive effects of metylphenidate, but it can only be considered as an exploratory study due to the very short length of the treatment -one week-, the small sample size and the crossover design with lack of wash-out period between treatments.

In relation to L-acetylcarnitine (LAC) a recently published study in children with FXS also diagnosed with ADHD was classified by the authors as a phase II study [13]. They found statistically significant stronger reductions of hyperactivity and improvement of social behaviour in patients treated with LAC, compared with the placebo group, as assessed by Conners' Global Index-Parents (CGI-P) and Vineland Adaptive Behavior Scales - Survey Form (VABS). LAC doses in that study ranged from 20 to 50 mg/kg/day. A previous smaller study carried out by the same research group assessed LAC at a much higher dose, 100 mg/kg/day and found significant statistical differences in favour of LAC in Conners' Abbreviated questionnaire for parents, but not for teachers [32]. Even though it is difficult to put value on the impact in the quality of life and social or intellectual performance of affected children of changes in scales assessed in a subjective way [31,36], the positive results observed with LAC merit replication, with appropriate samples sizes in children with FXS and ADHD, since no significant side-effects in the LAC group were reported.

However the mechanisms underlying the pathogenesis of FXS still remain unclear and the wide variety of treatments under evaluation probably reflects a lack of a single underlying pharmacological mechanism for the several impairments and symptoms associated with FXS. Several other medications (e.g., fenobam, lithium, aricept, memantine) that have been evaluated in open-label non comparative studies are now possible candidates for clinical controlled trials [11,37-40]. Other products (e.g. minocycline, alpha-tocopherol, melatonin) that have been being tested recently in animal models could open new prospects for future treatments [41-43].

Nevertheless given that intellectual, behavioural, emotional and/or learning performance in people with FXS is strongly influenced by different social factors, pharmacological treatments must be understood and assessed in the context of other interventions being in place. Often proposed areas of non pharmacological interventions include modifications in the home environment, more-tailored behavioural interventions and classroom environments, language and occupational therapy, and attention to social factors. Unfortunately, as a recent review points out, few studies have been published on the effectiveness of behavioural or social interventions among patients with FXS [11,44-46]. So it is advisable to promote studies that assess the effect of combined pharmacological and non-pharmacological interventions. In particular, it should be tested whether combined treatments could be more beneficial if administered in the early years in children with FXS.

Compared to previous reviews on pharmacological interventions on FXS, this review is the first one done following standardized methodology for systematic reviews. It also provides readers with complete data on the main characteristics of the studies, including aspects relating with their methodological quality, and their results.

## Conclusion

There is no robust evidence to support recommendations on pharmacological treatments in people with FXS in general or in those with an additional diagnosis of ADHD or autism. Available data are insufficient to draw firm conclusions or to make recommendations. Some positive results found in various studies should be confirmed by further parallel randomised trials, properly designed and with adequate statistical power, before these treatments could be translated to standard medical practice. New trials should include affected women and test whether pharmacological treatments could be more beneficial if administered in the early years in affected children, either alone or combined with non-pharmacological interventions.

## Competing interests

The authors declare that they have no competing interests.

## Authors' contributions

JRR and MIT conceived the study and participated in its design and coordination. JRR and JB performed literature search, analysis and data extraction from published studies. JRR, JB and MIT have contributed to discussion and conclusions and have taken part in the drafting of the final document. All authors have read and approved the final manuscript.

## Pre-publication history

The pre-publication history for this paper can be accessed here:


